# Local Use-Dependent Sleep in Wakefulness Links Performance Errors to Learning

**DOI:** 10.3389/fnhum.2018.00122

**Published:** 2018-04-03

**Authors:** Angelica Quercia, Filippo Zappasodi, Giorgia Committeri, Michele Ferrara

**Affiliations:** ^1^Department of Neuroscience, Imaging and Clinical Sciences, G. d'Annunzio University of Chieti-Pescara, Chieti, Italy; ^2^Institute for Advanced Biomedical Technologies (ITAB), G. d'Annunzio University of Chieti-Pescara, Chieti, Italy; ^3^Department of Biotechnological and Applied Clinical Sciences, University of L'Aquila, Coppito, Italy

**Keywords:** delta activity, errors, high-density EEG, overlearning, time on task, wayfinding

## Abstract

Sleep and wakefulness are no longer to be considered as discrete states. During wakefulness brain regions can enter a sleep-like state (off-periods) in response to a prolonged period of activity (local use-dependent sleep). Similarly, during nonREM sleep the slow-wave activity, the hallmark of sleep plasticity, increases locally in brain regions previously involved in a learning task. Recent studies have demonstrated that behavioral performance may be impaired by off-periods in wake in task-related regions. However, the relation between off-periods in wake, related performance errors and learning is still untested in humans. Here, by employing high density electroencephalographic (hd-EEG) recordings, we investigated local use-dependent sleep in wake, asking participants to repeat continuously two intensive spatial navigation tasks. Critically, one task relied on previous map learning (Wayfinding) while the other did not (Control). Behaviorally awake participants, who were not sleep deprived, showed progressive increments of delta activity only during the learning-based spatial navigation task. As shown by source localization, delta activity was mainly localized in the left parietal and bilateral frontal cortices, all regions known to be engaged in spatial navigation tasks. Moreover, during the Wayfinding task, these increments of delta power were specifically associated with errors, whose probability of occurrence was significantly higher compared to the Control task. Unlike the Wayfinding task, during the Control task neither delta activity nor the number of errors increased progressively. Furthermore, during the Wayfinding task, both the number and the amplitude of individual delta waves, as indexes of neuronal silence in wake (off-periods), were significantly higher during errors than hits. Finally, a path analysis linked the use of the spatial navigation circuits undergone to learning plasticity to off periods in wake. In conclusion, local sleep regulation in wakefulness, associated with performance failures, could be functionally linked to learning-related cortical plasticity.

## Introduction

The local use-dependent sleep theory proposes that sleep and wakefulness might coexist in the same time and in different areas of the brain (Krueger et al., 2008; Nobili et al., 2012; Siclari and Tononi, 2017). Ultimately, sleep and wakefulness might be considered dynamic processes that unfold through *local* brain changes from full wakefulness to global sleep and vice versa, as supported by growing data (Huber et al., 2004; Magnin et al., 2010; Ferrara and De Gennaro, 2011; Marzano et al., 2011, 2013; Sarasso et al., 2014).

Recently, it has been evidenced that during extended waking the progressive slowing of brain rhythms was due to *local use-dependent sleep* (Vyazovskiy et al., 2011; Hung et al., 2013; Bernardi et al., 2015; Nir et al., 2017), namely a type of sleep that occurs locally as the homeostatic consequence of a sustained use of neuronal circuits (Krueger et al., 2008, 2013; Krueger and Tononi, 2011). Specifically, it has been evidenced that in rats and in epileptic patients, delta/theta (2–6 Hz) waves progressively increased, triggered locally from use-dependent factors, such as synaptic overload in task-related circuits, and matched with a progressive decrease in task performance (Vyazovskiy et al., 2011; Nir et al., 2017). Similarly, in healthy subjects during prolonged waking experiments (>24 h), local task-dependent electroencephalographic (EEG) power changes in the 5–9 Hz range have been shown in task-related regions and associated with a worsening in performance (Hung et al., 2013; Bernardi et al., 2015).

The progressive decline in neurobehavioral performance, known as *time-on-task effect*, has been claimed as an expression of local use-dependent sleep, as homeostatic consequence of a sustained use of neuronal circuits engaged in a given task (Van Dongen et al., 2011). At biochemical level, the time-on-task effect is based on the ATP-cytokine-adenosine mechanism, that explains how the repeated and intense waking neuronal activity promotes sleep locally through the enhancement of sleep regulatory substances (Krueger et al., 2008, 2013), many of which are involved in NREM synaptic plasticity, specifically in the increase of slow wave activity (SWA, 0.5–4.5 Hz). Since SWA is homeostatically regulated at local level in those neuronal circuits subjected to plastic changes due to learning tasks, it has been considered as a specific index of *use-* and *learning*-dependent plasticity (Huber et al., 2004, 2006; Vyazovskiy et al., 2008). Specifically, the off-periods of SWA, namely periods of neuronal silence, were associated in sleep to the renormalization of synaptic strength, that allows memory consolidation processes during NREM sleep (Vyazovskiy et al., 2008, 2009, 2011; Tononi and Cirelli, 2014). In the aformentioned studies on local sleep in wake, periods of neuronal silence (off-periods) of individual slow waves were associated with performance errors (Vyazovskiy et al., 2011; Nir et al., 2017). According to Tononi and Cirelli (2014), local off periods in wake and sleep cannot be distinguished from each other and both could be the result of synaptic overload due to intense wake plasticity (Tononi and Cirelli, 2014; Vyazovskiy and Faraguna, 2015). Therefore, during wakefulness, local use-dependent sleep could be linked to learning-dependent plasticity.

However, so far, human studies that investigated local sleep in wakefulness (Hung et al., 2013; Bernardi et al., 2015; Muto et al., 2016; Nir et al., 2017) did not focus on time-on-task effects but rather on sleep restriction/deprivation effects on the neurobehavioral performance and, above all, did not explicitly manipulate learning.

**Figure 1:**
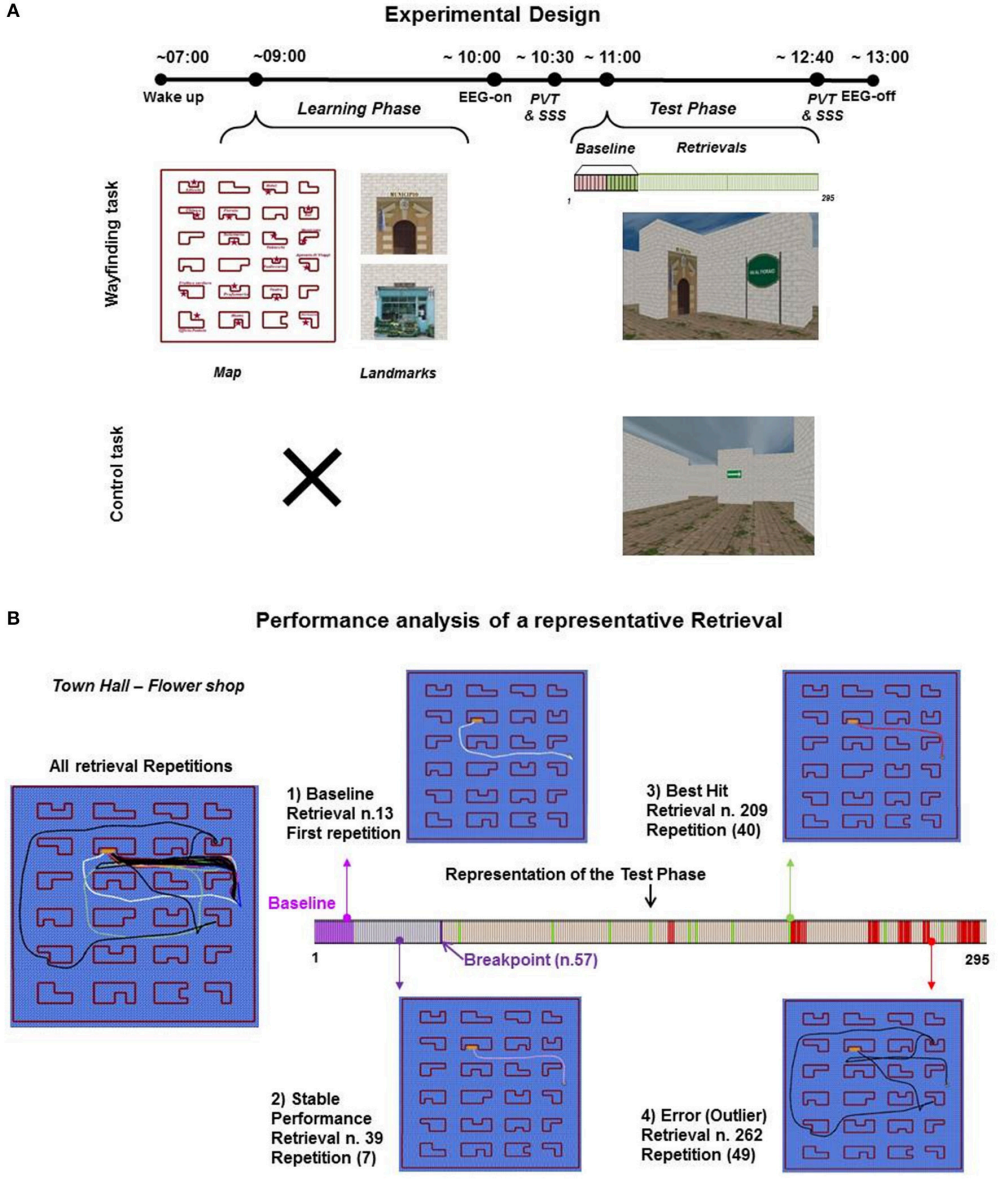
Experimental design and performance analysis of a representative retrieval. **(A)** In Wayfinding experiment (*n* = 20), participants woke up at ~7:00 and started the Learning phase (learning of the cognitive map with landmarks) at ~08:30. At ~11:00, participants performed the Test phase, retrievals to be completed, for ~2 h (example retrieval “Town Hall-Flower Shop”). Vertical bars refer to all retrievals presented (from 1 to 295): lilac bars = 8 baseline retrievals, green bars = the 8 retrievals of the baseline to be repeated, light green bars = the 8 retrievals of the baseline (light green) continuously repeated. The Control task (*n* = 9) was scheduled as the Wayfinding experiment, except for the Learning phase, so at ~11:00 participants performed the task (arrows to be followed) for ~2 h. At the beginning (~10:00) and at the end (~12:40) of each Test Phase, 10-min psychomotor vigilance task (PVT) and subjective sleepiness evaluation (Stanford Sleepiness Scale, SSS) were scheduled. In each experiment, participants underwent a continuous video hd-EEG recording. **(B)** The performance of a single subject on “Town Hall – Flower Shop” retrieval is showed through the relative graphic layouts: all repetitions traces; (1) the trace of the baseline retrieval (*n* = 13, repetition *n* = 1); (2) the trace of the stable performance (*n* = 39, repetition *n* = 7); (3) the trace of the best hit (*n* = 209, repetition *n* = 40); (4) the trace of an Error (*n* = 262, repetition *n* = 49). Arrows indicate the position of the relative trace along the Test phase, represented by a horizontal bar. Vertical bars refer to all retrievals of Wayfinding task (from 1 to 295): lilac bars = baseline retrievals, light lilac bars = retrievals before the breakpoint (violet horizontal bar, *n* = 57), peach colored bars = hits (green bars = best hits), red bars = outlier retrievals.

In order to investigate whether performance impairments could be the result of local sleep episodes induced by the overload in *learning*-related circuits, in the absence of previous sleep loss, in the present study we used a paradigm that combined overlearning (Shibata et al., 2017) and time-on-task effect, by focusing on delta activity and individual delta waves as specific indices of *use-* and *learning*-dependent plasticity. Specifically, we recorded high density electroencephalography (hd-EEG) during an intensive learning-related spatial navigation task, that consisted in the (hyper) use of a previously learned cognitive map (i.e., the mental representation of an environment with its landmarks) in order to reach different target locations (Wayfinding) without rest breaks (Figure 1A). Moreover, we compared the EEG changes of the Wayfinding experiment with those induced by a Control experiment, in which participants passively navigated within landmark-free environments by following arrows (i.e., without using a learned cognitive map), again unceasingly (Figure 1A).

We expected to observe, in brain regions involved in spatial navigation, off-periods of local sleep in wake during the task that required learning (Wayfinding experiment) but not during the task without learning (Control experiment). Moreover, off-periods were expected to be specifically linked to performance errors, as previously observed (Vyazovskiy et al., 2011; Nir et al., 2017).

## Materials and methods

### Subjects

Twenty healthy participants completed the Wayfinding experiment (all right-handed nonsmoker males; age range = 20–30 years, mean ± SD: 23.7 ± 1.7 years). After a month, 9 out of 20 participants took part in a Control experiment. No statistical methods were used to predetermine sample sizes, but our sample size is similar to those reported in previous studies (Hung et al., 2013; Bernardi et al., 2015). To avoid any kind of sleep debt and alterations of the sleep-wake cycle: (1) all selected participants reported no history of sleep, medical or psychiatric disorders and a good sleep quality (sleep schedule of 7–8 h/night), as assessed by self-rating questionnaires (Supplementary Table 1, Horne and Ostberg, 1976; Spielberg et al., 1983; Vignatelli et al., 2003; Violani et al., 2004; Sica et al., 2006; Curcio et al., 2013) (2) exclusion criteria included shift workers, athletes and participants that had traveled crossing time zones in the 3 months before the study. All participants reported experience with three-dimensional (3D) virtual environments, good spatial orientation skills (Wolbers and Hegarty, 2010) as assessed by a self-rating questionnaire (Pazzaglia et al., 2000, Supplementary Table 1) and underwent a practical test in a virtual environment to rule out inefficient motor skills during navigation and dizziness. Moreover, males outside the specified age range and females were excluded. The age and gender selection criteria are due to the fact that both factors are known to affect sleep, wayfinding and learning (Coluccia and Louse, 2004; Ohayon et al., 2004; Kurth et al., 2010). In particular, SWS (slow wave sleep) changes markedly across the lifespan (Ohayon et al., 2004; Kurth et al., 2010). Furthermore, males outperform females on wayfinding tasks, especially within complex environments (Coluccia and Louse, 2004). During recruitment, 12 subjects were discarded because they matched one or more exclusion criteria (see below and Supplementary Table 1). Two more subjects were discarded because they were not able to perform the Learning phase of the Wayfinding task (see next paragraph).

All participants underwent an afternoon experiment (Nap, Quiet or Active Wake session), whose aims go beyond the purpose of the present study. All participants signed an informed consent in accordance with the Declaration of Helsinki and were paid for their participation. The study was approved by the ethics committee for biomedical research of “G. D'Annunzio University” of Chieti-Pescara.

### Wayfinding experiment

Participants (*n* = 20) performed a spatial navigation task with explicit learning, that is a Wayfinding task (Figure 1A). The task was designed on the basis of the Cognitive Map Test (CMT) (Iaria et al., 2007), that assesses specific aspects of human topographical orientation. Briefly, the task required to orient oneself within a virtual environment containing different landmarks by first forming a mental representation of it, namely a cognitive map (Learning phase), and then using that mental representation to find the best way from one landmark location to another (Test phase).

Participants arrived at ~08:30 a.m. to check sleep diaries/actigraphy logs (see section Vigilance Performance and Subjective Sleepiness), and at ~9:00 a.m. began the Learning phase of the task, to avoid the influence of sleep inertia on learning (Ferrara and De Gennaro, 2000). The Learning phase of the task required participants to explore the city (24 buildings) to create its cognitive map, locating the 16 landmarks (shops and public buildings), whose pictures were shown to participants and named one by one by the experimenter before the learning phase. All the participants started the task at the center of the virtual environment facing the Pastry Shop and were first allowed ~15 min of free exploration (mean duration ± SD = 14.58 min ± 3.02), based on the results of a pilot study (Supplementary Table 2). The accuracy of the formed cognitive map was then evaluated by asking the participants to indicate the respective location of each landmark on a sheet of paper, on which the top-view outline of the city map was depicted. Participants explored the city for further sessions of ~5 min each (second map: mean duration ± SD = 5.18 min ± 3.07; third map: 4.92 min ± 2.06) until they reached an accuracy of 100% for two consecutive maps. The mean duration of the Learning phase was 24.69 min (SD: 5.05).

At ~11:00 a.m. participants performed the Test phase of the Wayfinding task (Figure 1A) that required to travel through the learned city from a landmark to another, following the shortest pathway and as quickly as possible. This phase comprised 16 types of retrievals, each characterized by a different route (from a landmark to another). In each of these retrievals, participants started facing a landmark and a sign, which reported the landmark they had to reach (Figure 1A). The Test phase was scheduled as follows: participants were first asked to complete the 16 retrievals type (EEG Baseline, see EEG Recording and Analysis), then half of them (the last 8) were repeated continuously in a randomized order, for a total of 279 retrievals until the end of the task, in order to obtain an hyper-use of the involved cerebral circuits and, at the level of each single repeated retrieval, to reach a performance optimization/stabilization useful to detect possible use-dependent errors. The Test phase was performed without rest, namely the retrievals followed one another without any kind of interruption (e.g., black screen), according to the time-on-task effect model proposed by Van Dongen and coworkers (Van Dongen et al., 2011). The Test phase was measured continuously with video hd-EEG recordings (128 electrodes, Electrical Geodesics, version 1.1) and lasted for ~2 h (mean ± SD: 1 h 44 min ± 8.00).

### Control experiment

After a month, we were able to collect further data on 9 out of the 20 participants of the Wayfinding experiment, to perform a Control task in order to distinguish effects of no interest (motor, perceptual and mere vigilance effects) from the learning-related effects. The Control task indeed required participants to navigate within virtual environments that resembled that of the Wayfinding task, with buildings with the same texture but without landmarks, therefore participants had not to learn any cognitive map. In fact, to complete the routes participants had only to follow the directions indicated by the green arrows on the walls along the routes, namely turn right, turn left, or move forward until the next sign was reached (Figure 1A). Also in this case virtual environments followed one each other without rest, as during the Test phase of the Wayfinding task.

For the Control task (Figure 1A), that did not require a learning phase, participants were asked to arrive at ~9:30 a.m. to check sleep diaries/actigraphy logs and caffeine's questionnaire (see Vigilance and Subjective Sleepiness), then participants were prepared for continuous video hd-EEG recordings. Control experiment lasted as long as the Test phase of the same participants (n = 9) of the Wayfinding experiment (mean duration ± SD: 1 h 41 min ± 9.09 and 1 h 45 min ± 6.20, respectively).

### Virtual environments

The virtual environments have been created by a freely available 3D software, Maze Suite (Ayaz et al., 2008), which enables to collect different qualitative and quantitative behavioral measures of the navigational performance. Each element present in the environment has a coordinate system: x z for what is placed on the ground and a y coordinate for the axis perpendicular to the floor. Participants navigated by using three different key-buttons (the upward, leftward, and rightward arrows available on the computer keyboard) to move in three different directions (left, right, forward). The software enables to measure participant's path (graphic layout), time and spatial units to completion of each path, to load multiple overlapping paths and to show a video of the traveling activity of a participant during the specific selected path.

### Vigilance and subjective sleepiness

For 1 week before each experiment (Wayfinding and Control), participants were asked to maintain a regular sleep-wake schedule, verified by detailed sleep diaries and a wrist-worn actigraph [wActiSleep+, ActiGraph, Pensacola, FL; sleep/wake algorithm (Cole et al., 1992; Sadeh et al., 1994)]; specifically, a Sleep Efficiency greater than 85% was required for either the week (average) and the night before each experiment to proceed with both experiments, to avoid any kind of sleep debt (for details, see Supplementary Table 3). Moreover, for 1 week before each experiment, the intake of caffeine-containing beverages, alcohol, medications and food that could affect the sleep-wake cycle was recorded by participants in a diary, based on the Stanford Caffeine Questionnaire (Nova et al., 2012). Those substances were forbidden from the day before and throughout each experiment. The night before each experiment, participants were asked to go to bed at their usual bedtime and to wake up at ~7:00 a.m. To evaluate vigilance at the beginning and at the end of each experiment (Wayfinding and Control, Figure 1A), participants performed a 10-min psychomotor vigilance task (PVT) (Dinges and Powell, 1985; Basner and Dinges, 2011), during which video hd-EEG was continuously recorded. Before and after each PVT session, subjective sleepiness was rated using the Stanford Sleepiness Scale (SSS) (Hoddes et al., 1973), a self-rating questionnaire ranging from 1 (“feeling active, vital, alert or wide awake”) to 7 (“no longer fighting sleep, sleep onset soon; having dream-like thoughts”).

### Sleep analysis

Sleep-wake schedule data of the week before each experiment were collected by actigraph and stored as the sum (activity) of 30 s intervals. These data were analyzed with Actilife (v.6.7.1, Actigraph, Pensacola, FL), using a sleep/wake detection validated algorithm (Cole et al., 1992; Sadeh et al., 1994). Bed and rise times from the questionnaire helped to frame the time in bed during which actigraphy data were analyzed.

### Behavioral analyses

In order to identify errors committed during the Wayfinding task, we measured the participants' paths (both as graphic layouts and as video recordings), as well as the time and units to completion. A performance analysis of a representative retrieval was shown in Figure 1B. We classified as errors those retrievals in which participants resulted as outliers in time and units respect to their average performance or, as shown by the graphic layout, the paths significantly deviated from the usual ones (detour), after the achievement of a stable performance. The stability of performance was evaluated with a trend analysis, carried out using Strucchange R-package (Zeileis et al., 2002). Due to the differences in the retrievals paths length, the trend analysis was based on the standardized values of time and units of the retrieval temporal series. This analysis allowed to estimate the so-called breakpoints (change-points), which indicate an interruption in the trend of a temporal series, in our case a change in spatial navigation performance, which results in a performance stabilization, likely due to the many repetitions of the same path. For each participant the first breakpoint was evaluated considering both time and units. To choose the retrieval after which the Wayfinding task performance was stabilized, the farthest retrieval between the two breakpoints (for time and units) from the beginning of the relative temporal series was chosen (Supplementary Figure 1). Only those outliers that occurred after this retrieval have been classified as errors for further analyses. To identify possible errors also during the Control task, for each virtual environment we measured the number of arrows in which participants made a mistake turning in the wrong direction. Each potential error was checked in the video recording before being included in the analyses to exclude closure of eyelids and episodes of looking away from the screen (distractions).

Performance improvement on each retrieval type was calculated as the percentage of deviation units from the relative optimal path, that corresponds to the Euclidean distance between the start point (coordinates x_1_ z_1_) and the endpoint (coordinates x_2_ z_2_) of each retrieval type, according to the subsequent formula:

(1) $$\text{Optimal path}=\sqrt{{\left({\text{x}}_{1}-{\text{x}}_{2}\right)}^{2}+{\left({\text{z}}_{1}-{\text{z}}_{2}\right)}^{2}}$$

Since the Euclidean distance cannot be reached, the improvement has a negative sign. The deviation from the optimal path was calculated for each subject as the mean percentage change of the mean deviation units from the relative optimal paths across the retrievals, according to the subsequent formula:

(2) $$\text{Deviation}=\frac{\;1}{\text{N}}\sum_{\text{i}=1}^{\text{N}}\frac{\left(\text{Optimal Path }{\text{Units}}_{\text{i}}-\text{ Retrieval }{\text{Units}}_{\text{i}}\right)}{\text{Optimal Path }{\text{Units}}_{\text{i}}}*100$$

with N as the number of considered retrievals.

### EEG recording and analysis

Analysis of wake EEG was carried out on both experiments (Wayfinding and Control) and on PVT recordings. Hd-EEG signals were collected continuously during both tasks, using a sampling frequency of 250 Hz, and referenced to the Cz electrode. Skin/electrode impedances were kept <50 kΩ at the beginning of each recording session. The position of each electrode and anatomical landmarks (pre-auricolar points, nasion, vertex) were digitized by a 3D digitizer (Polhemus, 3Space Fastrak). EEG data were processed off-line. Data were band-pass filtered (Butterworth second order filter, 0.5–45 Hz) and each recording was visually inspected to identify channels and epochs containing artifacts. Rejected channels were then interpolated using spherical splines (NetStation, Electrical Geodesic Inc.). Analysis was carried out using Matlab (Mathworks Inc., Natick, MA, v. 7.10 2012). Independent component analysis (ICA) was used to remove ocular, muscle, and electrocardiographic artifacts (Barbati et al., 2004). Only ICA components with specific activity patterns and component maps characteristic of artefactual activity were removed. After excluding electrodes located on the neck/face region, the signal for each channel was down-sampled to 125 Hz and re-referenced to the average of the remaining good channels, >100 channels per recording (Lustenberger and Huber, 2012). For each EEG derivation, Power Spectral Density (PSD) estimates were computed by the Welch method, based on fast Fourier transform (FFT) in 4 s Hamming nonoverlapping periods, which were obtained by EEG signals for each retrieval starting from the landmark presentation. PSD estimates were thus performed with a 0.25 Hz bin resolution. In the Wayfinding experiment, EEG windows of retrievals were differently grouped on the basis of behavioral performances, i.e., errors or hits, in the same two equal temporal intervals from the breakpoint retrieval detected in the behavioral analysis. In this way, four different PSD estimates were obtained: two for retrievals in which errors occurred (one in the first and one in the second temporal intervals) and two for hits (one in the first and one in the second temporal intervals). The first 16 retrievals were taken into account to estimate the Wayfinding baseline PSD. To compare Wayfinding and Control experiments, EEG windows were grouped in four equal temporal intervals from the breakpoint time of the Wayfinding task for both tasks. As for Wayfinding task, four different PSD estimates were obtained from retrievals (errors and hits combined) of each temporal interval. As for Control task, four different PSD estimates were obtained from routes of each temporal interval. To make the Baseline of the two tasks comparable, for each participant, PSD estimate of Control Baseline was calculated in the same time interval of the Wayfinding baseline. For all the experimental conditions described above, power in delta and theta bands for each EEG channel was then calculated as the average of PSD values in the 1–4 Hz and 4.25–7.5 Hz frequency intervals respectively. Delta and theta values were log transformed. As for the Wayfinding experiment, delta and theta bands were calculated for errors and hits in the first and second temporal intervals and compared with the Wayfinding baseline (delta and theta bands of the first 16 retrievals), see Statistical Analyses for details. Moreover, for both Wayfinding and Control experiments, delta and theta bands were calculated for each quartile and contrasted with the delta and theta power of the Baseline of Wayfinding and Control experiment, see Statistical Analysis for details. On the basis of our experimental hypothesis, delta power was chosen as specific index of use- and learning-dependent plasticity in wake, as classically found in sleep (Huber et al., 2004, 2006; Vyazovskiy et al., 2008), while theta power (5–9 Hz) was chosen as the wake EEG marker of sleepiness (Finelli et al., 2000, 2001; Strijkstra et al., 2003; De Gennaro et al., 2007). Moreover, both theta and delta activity are claimed as the neural correlates of spatial navigation ability (Watrous et al., 2011, 2013; Jacobs, 2013; Ekstrom and Watrous, 2014; Vass et al., 2016).

Furthermore, an analysis of the individual delta waves was performed on errors and hits of the Wayfinding task. In fact, performance errors during wake are associated with the negative peak of individual delta waves, that correspond to periods of neuronal silence (Vyazovskiy et al., 2011). In detail, EEG signals for each derivation were re-referenced to the average of the 2 mastoids and low-pass filtered at 4 Hz using a Chebyshev type II filter. Filter parameters were selected in order to achieve minimal wave shape and amplitude distortion. For each channel, individual half-waves were detected on artifact-free 4 s epochs. Half-waves were defined as negative deflections between 2 consecutive zero crossings. The position of the negative and positive peaks was determined based on the zero crossings of the signal. To avoid possible confounding effects due to spurious low amplitude deflection of the signal, all 1–4 Hz detections were subdivided into 5 equal percentiles based on their negative peak amplitude. The distribution was determined by pooling all 1–4 Hz detections across each retrieval of the Wayfinding task, for each subject and channel separately, standardizing for the length of each retrieval; only the detections that exceeded the top 20% amplitude threshold were considered delta waves and selected for further analysis. This detection procedure was similar to those used in previous studies (Riedner et al., 2007; Hung et al., 2013; Bernardi et al., 2015).

### Source localization

To localize the sources of brain activity, a current density analysis in 3D MNI space was performed, using the exact low-resolution brain electromagnetic tomography, eLORETA, in the frequency domain (Pascual-Marqui, 2007). The current source density distribution of delta and theta frequency band power was estimated on a grid of 6,239 voxels, with a spatial resolution of 5 mm for each experimental condition. Specifically, for the Wayfinding experiment, delta and theta power values of errors and hits in the first and second temporal intervals (the two equal temporal intervals from the breakpoint retrieval detected in the behavioral and EEG analysis) were evaluated for each voxel and compared with the corresponding values of Wayfinding baseline (the first 16 retrievals). For both Wayfinding and Control experiments, delta and theta bands power values of the fourth temporal interval (the fourth of the equal temporal intervals from the breakpoint time of the Wayfinding task) were evaluated for each voxel and compared with the relative Baseline (Wayfinding and Control), calculated in the same time interval of the Wayfinding baseline for each participant. Cortical maps of the differences between the conditions described above were finally obtained. Only the voxels whose values exceeded the 95% percentile of the value distribution were considered to individuate cortical regions of significant activity. For each region, the coordinate of the voxel corresponding to the maximum value, as well as the coordinate of the baricenter was found (Table 1).

**Table 1:** Cortical regions of significant activity (differences between the conditions).

	**Anatomical region (MNI coordinatates)**						
	**Maximum (mm)**				**Baricenter (mm)**		
	***x***	***y***	***z***		***x***	***y***	***z***
**WAYFINDING TASK vs. BASELINE (SECOND HALF)**							
**Delta power difference–errors**							
Left precuneus	−30	−85	40	Left Superior Parietal Lobule	−37	65	46
Left middle frontal gyrus	−45	30	40	Left middle frontal gyrus	−39	26	38
Right precentral gyrus	50	−5	55	Right precentral gyrus	48	−3	55
**Delta power difference–hits**							
Left precuneus	−38	85	40	Left Superior Parietal Lobule	−39	−59	48
Left middle frontal gyrus	−45	30	40	Left middle frontal gyrus	−39	29	35
Right precentral gyrus	45	−5	53	Right precentral gyrus	47	−4	57
**Theta power difference–errors**							
Left inferior parietal lobule	−60	−39	41	Left inferior parietal lobule	−54	−44	41
Right precentral gyrus	50	−5	55	Right Precentral Gyrus	49	−5	53
**Theta power difference–hits**							
Left inferior parietal lobule	−60	39	41	Left inferior parietal lobule	−52	−46	39
Right Precentral Gyrus	50	−5	55	Right Precentral Gyrus	50	−11	49
Left inferior frontal gyrus	−55	15	30	Left inferior frontal gyrus	−51	4	38
**WAYFINDING vs. CONTROL TASK (FOURTH QUARTILE)**							
**Delta power difference**							
Left cuneus	−25	−90	35	Left precuneus	−30	−75	41
Right superior parietal lobule	32	−80	40	Right precuneus	32	−80	40
Right precentral gyrus	20	−20	70	Left Superior frontal gyrus	−1	−7	68
Right primary sensory-motor cortex	35	−25	70	Right primary sensory-motor cortex	30	−32	67

### Statistical analyses

Data analysis was performed via R-coded scripts (R version 3.3.1), using R packages (R Core Team, 2013). Outliers were defined as data points below Q1-1.5^*^IQR (interquartile range) or above Q3+1.5^*^IQR. Outliers influence diagnostics were performed (Mahalanobis distance, Leverage and Cook's distance) before data analysis. No subjects were excluded from the statistical analysis. Outliers influence was not applied to data known to be nonnormally distributed. Normality was formally tested (nortest package), nonparametric analyses were applied to nonnormally distributed data and to compare variables between groups that included less than 10 subjects. For the Wayfinding experiment (sample size = 20), variables of interest (hits and errors, as well as SSS scores and PVT metrics) were compared by parametric statistics (one-tailed *t*-tests, *P* < 0.05, Bonferroni corrected). Nonparametric Spearman's rho correlations (Bonferroni corrected) were performed between the Wayfinding experiment variables (number of hits and errors) and delta/theta band (source estimation). As for SSS score and PVT metrics of the Control experiment (sample size = 9), Wilcoxon Signed-rank tests, available on coin package (Hothorn et al., 2008), were performed. Finally, to test the difference in SSS score and PVT metrics between Wayfinding and Control experiments, we performed a nonparametric ranked analysis of covariance, RANCOVA (Feys, 2016), that was based on Residual rank scores, with residuals of the pre-test measures as covariate, the Group variable as the predictor in the regression with residuals of the post-test measures as dependent variable. First, ignoring Groups (Wayfinding and Control), the pre-test and post-test measures were ranked separately, then a regression was run of the post-test ranks on the pre-test ranks. Second, an ANOVA was carried out on the residuals obtained from the regression (residual rank scores).

Finally, effect size was computed (compute.es package) for the significant Wayfinding results; to evaluate the effect size of significant Wilcoxon Signed-rank tests, Exact Wilcoxon-Pratt Signed-rank (coin package) tests were performed.

Differences in hd-EEG activity were assessed using a cluster-based nonparametric randomization test, considering multiple-comparisons (Maris and Oostenveld, 2007). The test allowed to evaluate the topography of electrodes in which the experimental conditions showed a significant difference in delta or theta band. In detail, for each electrode the difference between the experimental conditions was tested by a dependent-sample *t*-value. All samples showing a *t*-value greater than a threshold corresponding to *p* = 0.05 were spatially clustered in connected sets on the basis of spatial and spectral adjacency. Cluster-level statistics were calculated by taking the sum of the *t*-values within every cluster. The maximum of the cluster-level statistics was taken to calculate the significance probability by Monte Carlo method: a reference distribution of maximum cluster *t*-values was obtained by randomizing data across two conditions for 5,000 repetitions to evaluate the statistics of the data. The test is freely available in FieldTrip (Oostenveld et al., 2011), an open-source Matlab toolbox.

A formal path analysis was performed using a structural equation modeling, implemented in Lavaan package (Rosseel, 2012), to examine interrelationships between the subsequent variables: (a) repetition-dependent improvement, calculated as deviation units from optimal path obtained before the first error, weighting for both the number of best hits reached before the first error (weighted average for each type of retrieval, with frequency of best hits as weights) and the number of retrieval repetitions before the first error (excluding the 16 test phase retrievals); (b) use-dependent saturation, corresponding to the temporal starting point of errors; (c) local sleep in wake (delta source activity of the second temporal interval of errors); (d) number of errors (number of errors of the second temporal interval). Specifically, the structural equation model tested was specified by three directional multiple regressions, to test whether errors were directly induced by local sleep in wake and indirectly influenced by use-dependent saturation and repetition-dependent improvement, through its interaction with use-dependent saturation (**Figure 5**):

**Table:** 

Dependent variable	~	Independent Variable
1 Use-dependent saturation	~	Repetition-dependent improvement
2 Local Sleep	~	Use-dependent saturation
3 Errors	~	Local Sleep

Validated metrics were used to test model fit (Rosseel, 2012), specifically: chi-square test statistic, that should not be significant for a good model; standardized root mean residuals (SRMR, good model lower than 0.06); root mean square error of approximation (RMSEA, good model lower than 0.05); comparative fit index (CFI), an index to compare the fit model and the independence model, that assumes uncorrelated variables and should be above 0.95 for a good model; Akaike's information criterion (AIC), to compare the model (the lowest is the best model) to a saturation model. Moreover, individual path coefficients were examined for significance.

## Results

### Behavioral data: time-on-task selectively increases both errors and hits during wayfinding task

**Figure 2:**
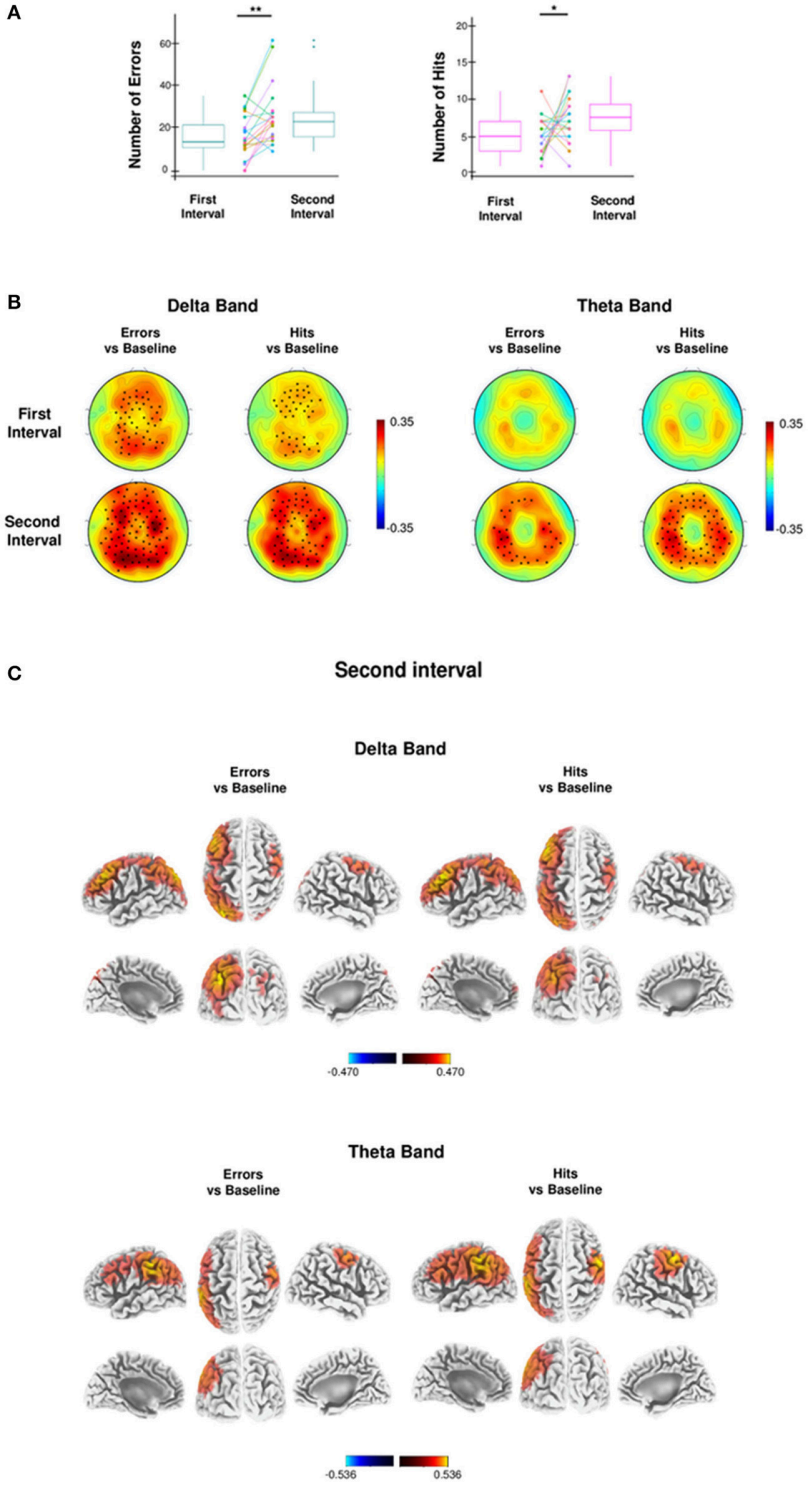
Wayfinding analysis. **(A)** Box plots and dot plots show the distribution of the first and the second half of errors and best hits. The box plots show: the 25th and 75th percentiles; the variability (whiskers); the median values (bold lines); the outliers (dots outside whiskers). Dot plots show the subjects paired values (colored dots) connected by the colored lines. The mean number of errors and hits in the second half compared to the first half increases significantly (one-tailed paired *t*-test, ^*^p < 0.05; ^**^p < 0.01). **(B)** Topographic distribution of absolute delta (1–4 Hz) and theta power (4.5–7.5 Hz), contrasting the first half (top) and the second half (bottom) of Errors and Hits with Baseline during the Test phase, using a nonparametric statistical test for multiple comparisons (*p* < 0.05, black stars indicate significant difference between conditions). **(C)** Brain sources of delta (top) and theta (bottom) power of the second half of Errors and Hits, compared with the respective brain sources of the Baseline, at voxel level. Values of bands power at voxel level were obtained by eLORETA (threshold 95% of the maximum value).

Performance analysis was mainly focused on errors that occurred during the Test phase of the Wayfinding task. A representative analysis of performance outcomes for a single retrieval is shown in Figure 1B. Errors were those retrievals in which participants resulted outliers and that occurred only after the achievement of a stable performance, namely after a breakpoint retrieval (Supplementary Figure 1; for details see Behavioral analyses). On average, the first breakpoint retrieval was reached at 22.87 min (SD: ± 9.12 min.), at retrieval number 62.5 (SD: ± 24.38), whereas the first error was made at 36.77 min. (SD: ± 15.28 min.), at retrieval number 104.80 (SD: ± 40.60). In general, participants made several errors (mean, SD: 39.45 ± 18.53; see a representative Error in Figure 1B and Supplementary Video 1). Errors were divided in two equal temporal halves from the breakpoint retrieval. We found a significant increase in the mean number of errors in the second temporal interval compared to the first [one-tailed paired *t*-test, *t*_(19)_ = 3.55, *p* = 0.0010, *d* = 1.12, Figure 2A]. In the same two temporal intervals of errors we then calculated the number of best hits (mean, SD: 14.65 ± 1.27), namely the minimum values—both in units and time—to complete each retrieval. Like errors, also the number of best hits significantly increased during the second temporal interval compared to the first [one-tailed paired *t*-test, *t*_(19)_ = 2.06, *p* = 0.0262, *d* = 0.65, Figure 2A]. Differently from the Wayfinding task, instead, the number of Errors during the Control task did not differ from the two equal temporal halves, as shown by Wilcoxon Signed-rank test (*W* = 42.0, *p* = 0.012, *r* = 0.54; *W* = 15.5, *p* = 0.46, respectively). Accordingly, the probability of making Errors during the Wayfinding task (total number of Errors/Numbers of Retrievals) was significantly higher than the probability of making Errors during the Control task (total number of Errors/total numbers of Arrows), as shown by Exact Wilcoxon-Pratt Signed-rank test (*z* = 2.66, *p* = 0.039, *r* = 0.61).

### Local increases of EEG delta power during the intensive wayfinding task are positively related to errors and negatively related to hits

Given the recent association between increased EEG delta/theta power and performance worsening during an extended task in both rats and humans (Vyazovskiy et al., 2011; Hung et al., 2013; Bernardi et al., 2015; Nir et al., 2017), we performed a topographic analysis of the time course of the EEG power changes in the same two equal temporal intervals from the breakpoint retrieval detected in the behavioral analysis. In particular, absolute delta (1–4 Hz) and theta (4.5–7.5 Hz) power during errors and hits were compared with the baseline (first 16 retrievals) delta and theta bands.

For both errors and hits, the nonparametric randomization test (*p* < 0.05, corrected) revealed a specific topography of EEG delta power that significantly increased both in the first and the second temporal interval with respect to baseline, with a progressive enhancement in significant electrodes engaged by the task (Figure 2B). Source localization showed that delta power increase was focused in the left parietal cortex, in the bilateral sensorimotor and premotor regions and in the left frontal region. In particular, considering only the voxels whose delta power difference values exceeded the 95% percentile of the distribution of values of both conditions (errors vs. baseline and hits vs. baseline), 3 cortical regions were identified (Figure 2C and Table 1): (1) in the left parietal cortex the delta power difference was spread out in the precuneus, inferior parietal lobule and post-central regions; (2) in the left frontal cortex the increase of delta power was maximally located in the middle frontal gyrus; (3) in the right frontal cortex the maximum was located in the precentral gyrus. All these regions are engaged in spatial navigation tasks (Boccia et al., 2014; Ekstrom et al., 2014; Slone et al., 2016).

The mean power differences of the 3 cortical regions were calculated and correlated with the mean number of errors and hits in the second temporal interval. We found a positive correlation (Bonferroni corrected) between the mean number of errors and the delta power increase of errors localized in the left parietal (rho = 0.49, p-corrected = 0.04128, *d* = 1.12) and right frontal (rho = 0.58, p-corrected = 0.0109, *d* = 1.42) cortex. By contrast, the mean number of hits of the second temporal interval was negatively correlated with the delta power increase of hits in the left frontal (rho = −0.54, p-corrected = 0.0204, *d* = 1.28) and right frontal cortex (rho = −0.49, p-corrected = 0.0405, *d* = 1.12).

In contrast to delta power, theta power of errors and hits significantly increased only in the second temporal interval compared to baseline, as revealed by the nonparametric randomization test (*p* < 0.05, corrected; Figure 2B). Source localization showed a theta increase for both errors and hits with respect to baseline in the left parietal and right frontal cortex (Table 1). Moreover, a theta increase for hits was observed also in the left frontal cortex (Table 1). The mean power differences of the 3 cortical regions were calculated and correlated with the mean number of errors and hits in the second temporal interval. We found a positive correlation (Bonferroni corrected) between the mean number of errors and the theta power increase of errors only in the right frontal cortex (rho = 0.55, p-corrected = 0.01080, *d* = 1.32). Moreover, the mean number of hits was negatively correlated with the theta power increase of hits only in the left parietal cortex (rho = −0.51, p-corrected = 0.03138, *d* = 1.19).

### Individual delta waves are related to errors during wayfinding task

**Figure 3:**
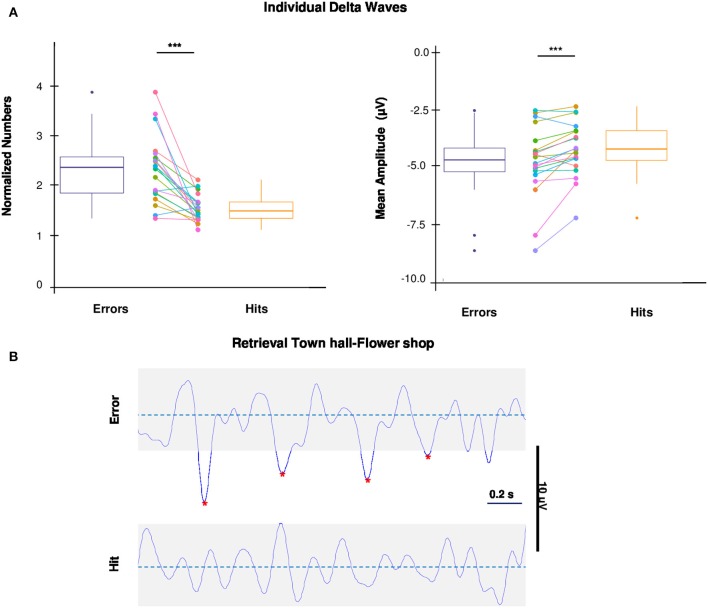
Individual delta waves and Wayfinding performance. **(A)** Box plots and dot plots show the distribution of number and amplitude of delta waves during errors and hits. The box plots show: the 25th and 75th percentiles; the variability (whiskers); the median values (bold lines); the outliers (dots outside whiskers). Dot plots show the subjects paired values (colored dots) connected by colored lines. The mean number and negative amplitude of delta waves during errors is significantly higher compared to hits (one-tailed paired *t*-test, ^*^p < 0.05; ^***^p < 0.001). **(B)** Representative examples of individual delta waves (raw traces) during an Error (top) and a Hit (bottom) of the same retrieval (“Town Hall–Flower Shop”) of a subject. Red stars indicate the negative peaks of delta waves detected.

To investigate the possibility that task-related errors are the result of periods of neuronal silence, reflected in individual delta waves (Riedner et al., 2007; Nir et al., 2011, 2017; Vyazovskiy et al., 2011; Hung et al., 2013; Bernardi et al., 2015), we compared the number and the amplitude (negative peak) of individual delta waves during errors and hits (for details see EEG Recording and Analysis). For each electrode covering the 3 above mentioned cortical regions (right and left frontal, left parietal), the number of delta waves and the relative amplitude were averaged across the electrodes of the cortical regions for both errors and hits and then compared. Both number and amplitude of delta waves were significantly higher during errors than hits [one-tailed paired *t*-tests, respectively: *t*_(19)_ = 5.1191, *p* = 0.00003, *d* = 1.62; *t*_(19)_ = −3.6765, *p* = 0.0008, *d* = 1.16; Figure 3A]. A representative EEG raw signal, recorded from one subject on the same channel (PO8), shows individual delta waves during an Error and a Hit of the same retrieval (Figure 3B).

### A *Learning* dependent increase of delta power differentiates between wayfinding and control task

**Figure 4:**
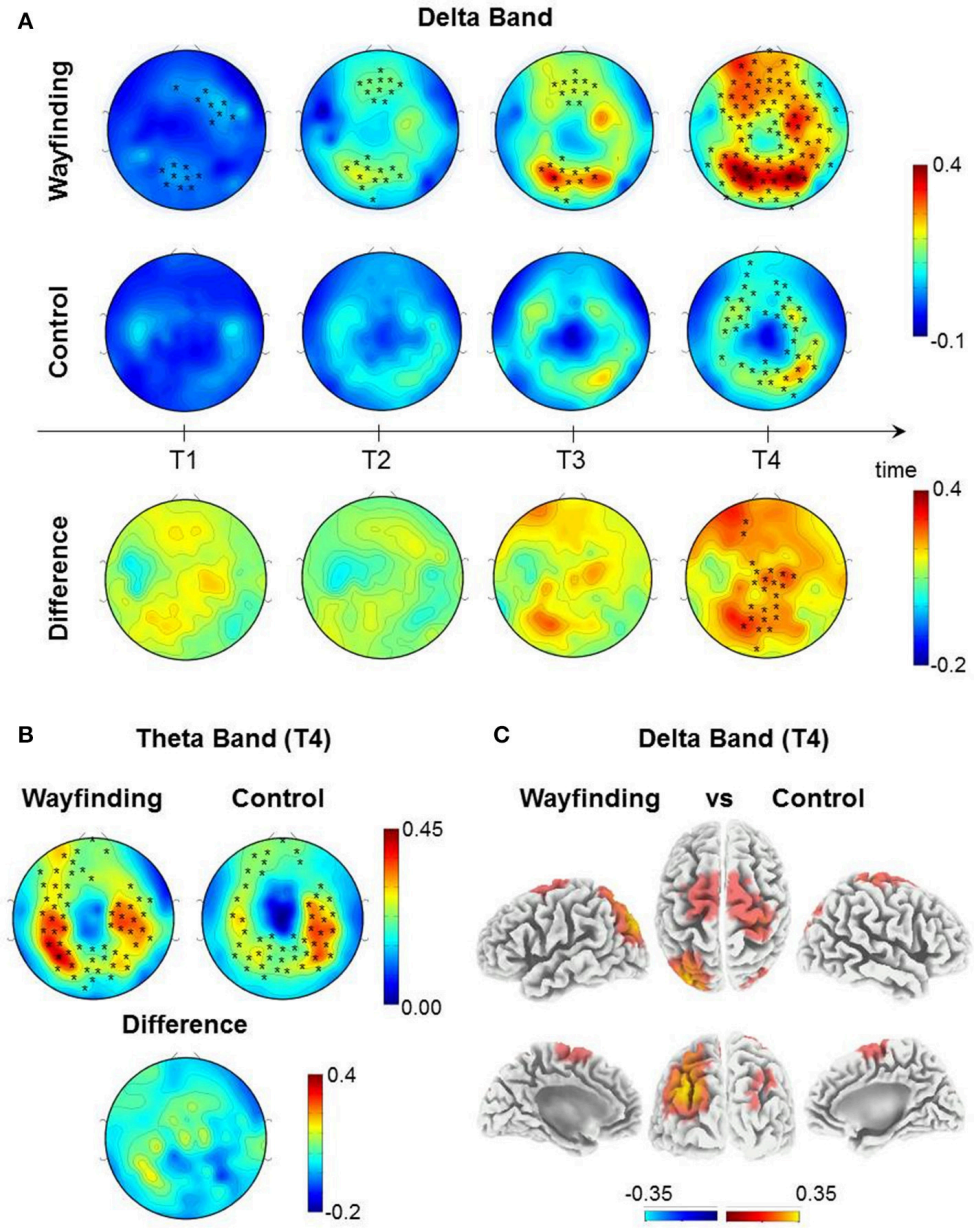
Comparisons between Wayfinding and control task. **(A)** Topographic distribution of absolute delta power (1–4 Hz), contrasting each quartile (T1–T4) of the Wayfinding task (first row), the Control Task (second row) with the respective Baseline and the difference of each quartiles between the tasks (third row). **(B)**Topographic distribution of absolute theta power (4.5–7.5 Hz), contrasting the last quartile (T4) of the Wayfinding task (left) and the Control task (right) with the respective Baseline and the difference between the task. Topographic distribution of bands power was obtained by nonparametric statistical test for multiple comparisons first line (*p* < 0.05, black stars indicate significant differences between conditions, 95% threshold) using a nonparametric statistical test for multiple comparisons (*p* < 0.05, black stars indicate the clusters of electrodes showing a significant increase in delta and theta power). **(C)** Brain sources of delta power of the difference between the last quartile (T4) of the tasks at voxel level. Values of bands power at voxel power were obtained by eLORETA (threshold 95% of the maximum value).

To assess the time course of delta and theta power during the Wayfinding and the Control tasks, both tasks were divided into four equal temporal intervals (quartiles) from the breakpoint time of the Wayfinding task. We contrasted absolute delta and theta power of each quartile with the corresponding Control task baseline (see EEG Recording and Analysis for details). The nonparametric randomization test (*p* < 0.05, corrected) revealed that during the Wayfinding task, delta power increased progressively across each quartile, specifically, from the first to the last quartile (Figure 4A, first row). Instead, during the Control task, delta power increased significantly only during the last quartile (Figure 4A, second row). Furthermore, delta power of the last quartile was higher during the Wayfinding task compared to the Control one (Figure 4A, third row). Source localization showed that this delta power difference was focused on the parieto-occipital and centro-frontal regions. In particular, considering only the voxels whose values exceeded the 95% percentile of the value distribution, the main difference was found in the left parieto-occipital cortex, as well as in the right parietal areas, in the right primary sensory-motor cortex and bilaterally in the frontal cortex (Figure 4C and Table 1).

For theta power, the nonparametric randomization test (*p* < 0.05, corrected) revealed a significant increase only during the last quartile of both tasks (Figure 4B), which did not differ between them.

### Wayfinding and control tasks did not differ in terms of vigilance

Before and after each task, we measured vigilance both subjectively by using the self-rating Stanford Sleepiness Scale (SSS) (Hoddes et al., 1973) and objectively by using a 10-min Psychomotor vigilance task (PVT) (Dinges and Powell, 1985). The former increased both after the Wayfinding task [one-tailed *t*-test: *t*_(19)_ = 6.328, p-corrected = 0.000002, *d* = 2] and the Control task (Exact Wilcoxon-Pratt Signed-rank test: *z* = 2.32, *p* = 0.02784, *r* = 0.82). Instead, objective vigilance did not differ before and after both tasks. In detail, the PVT metrics (Basner and Dinges, 2011), namely the number of lapses and the measures of psychomotor response speed (mean 1/RT and mean slowest 10% 1/RT) did not differ before and after the Wayfinding task, as shown by one-tailed paired *t*-test on number of lapses [*t*_(19)_ = 0.86, *p* = 0.1989], on mean 1/RT [*t*_(19)_ = −2.05, *p* = 0.9733] and on mean slowest 10% 1/RT [*t*_(19)_ = −1.92, *p* = 0.9615]. The same metrics did not differ also before and after the Control task, as shown by Wilcoxon Signed-rank test on number of lapses (*W* = 3, *p* = 0.18), on mean 1/RT (*W* = 7, *p* = 0.9727) and on mean slowest 10% 1/RT (*W* = 20; *p* = 0.6328).

Moreover, we assessed the difference between Wayfinding and Control task for SSS score and for PVT metrics. Pre-test scores did not differ between the tasks, as shown by Wilcoxon Signed-rank test on subjective sleepiness scores (*W* = 1.5, *p* = 0.18), on number of lapses (*W* = 6, *p* = 0.78), on mean 1/RT (*W* = 20, *p* = 0.82) and on mean slowest 10% 1/RT (*W* = 23; *p* = 1). More importantly, post-test scores did not differ [subjective sleepiness score: *F*_(1, 16)_ = 1.1514, *p* = 0.2992; number of lapses: *F*_(1, 16)_ = 0.8306, *p* = 0.3756; response speed: *F*_(1, 16)_ = 0. 053, *p* = 0.82; and mean slowest 10% 1/RT: *F*_(1, 16)_ = 0.059, *p* = 0.81], as assessed using a nonparametric ranked analysis of covariance (Feys, 2016) (RANCOVA) with the post-test as dependent variable predicted by group (Wayfinding and Control) and the pre-test as covariate (for details see Materials and Methods).

Finally, also the comparison of topographical EEG changes of absolute delta and theta power in the two PVT sessions following the Wayfinding and Control tasks did not show any significant difference.

### Path analysis: learning-related use-dependency predicts local sleep

**Figure 5:**
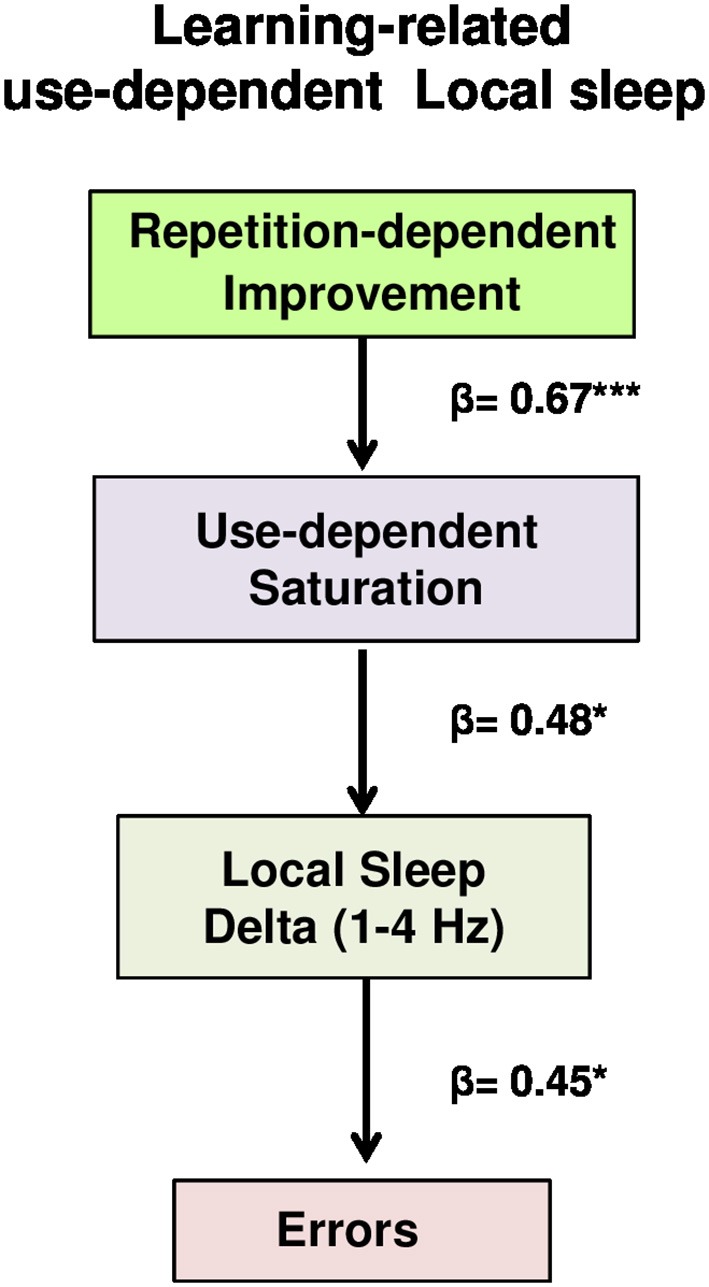
Learning-related use-dependent local sleep model. Path analysis model examining the contributions of repetition-dependent improvement and use-dependent saturation to local sleep and errors in a hypothesized model. Values represent standardized regression weights. The model fits were estimated (χ^2^ = 1.02, *p* = 0.79, RSMR = 0.041, RMSEA = 0.000, CFI = 1.00). Learning-related use-dependent local sleep model was better than saturated model (AIC = 213.247, AIC = 218.226, respectively). Solid line indicated significant paths. ^***^p < 0.001, ^*^ *p* < 0.05.

As we have seen, the effect of time-on-task on delta power was more prominent during the Wayfinding task than the Control one. In detail, the increase of errors during the wayfinding performance was specifically associated to delta waves, and are not due to basic/motor perceptual aspects nor to vigilance. However, these results did not explain how the progressive improvement during the Wayfinding task could lead to errors, as a consequence of local sleep in wake. To investigate how performance improvement and local sleep in wake interact to induce performance failures, we performed a path analysis, testing direct and indirect relationships between the subsequent factors (Figure 5, for details see Materials and Methods): (a) repetition-dependent improvement, calculated as deviation units from the optimal path (“equation 2,” see Materials and Methods for details), weighted for the number of best hits obtained before the first error and the number of repetitions before the first error (see Materials and Methods for details); (b) use-dependent saturation, namely the temporal starting point of errors; (c) local sleep in wake, namely delta source activity during the second temporal interval of errors, and (d) errors (number of errors of the second temporal interval). The path analysis, specified by three directional multiple regressions, tested whether performance improvement and its interaction with use-dependent saturation, that directly influenced local sleep in wake, indirectly induced errors. The model, called “learning-related use-dependent local sleep model,” provided a good statistical fit (for details, see Materials and Methods for details), specifically: nonsignificant chi-square test statistic (χ^2^ = 1.02, *p* = 0.79); standardized root mean residuals (SRMR) lower than 0.06 (SRMR = 0.041); root mean square error of approximation (RMSEA) lower than 0.05 (RMSEA = 0.000); CFI, namely the comparison between the fit model and the independence model (uncorrelated variables assumed), above 0.95 (CFI = 1.00). Furthermore, as indicated by the AIC, the learning-related use-dependent local sleep model resulted better than the saturated model (AIC = 218.226, 213.247; respectively). As shown in Figure 5, the three directional regressions demonstrated that: (1) repetition-dependent performance improvement predicted the temporal starting point of errors (β = 0.67, *p* = 0.000), (2) this use-dependent improvement-related factor (saturation), in turn, significantly predicted the amount of local sleep in wake (β = 0.48, *p* = 0.014), which ultimately (3) determined the number of errors (β = 0.45, *p* = 0.024).

## Discussion

In the present study, by using an *overlearning* paradigm without *rest breaks* (Shibata et al., 2017) during wayfinding, we induced off-periods of local sleep in wake indexed by the occurrence of individual delta waves in task-related regions, as shown by source localization. Interestingly, we found that off-periods were strictly and specifically linked to performance errors. By contrast, the intensive use of the same task-related circuits during a control experiment, that did not require learning, did not affect neither the behavioral performance nor the local sleep in wake, although vigilance did not differ between the tasks. Finally, by using a formal path analysis, our findings could support a framework in which performance failures are a consequence of the amount of local sleep in wake, which in turn depends on the intensive and repeated use of the spatial navigation circuits undergone to learning-dependent plasticity processes. The present study shed light on the factors triggering off periods of local sleep in wake and their behavioral consequences, namely performance errors, highlighting their tight relationship with use-dependent learning-related plasticity. These findings are discussed in detail below.

### Difference between wayfinding and control task

Although episodes of local sleep in wake could seem a maladaptive response inasmuch they have been linked to performance impairments, both in rats and humans (Vyazovskiy et al., 2011; Bernardi et al., 2015; Nir et al., 2017), it is likely that they could be involved in the initiation of local restorative processes, including synaptic homeostasis (Vyazovskiy et al., 2011; Tononi and Cirelli, 2014; Rodriguez et al., 2016). Indeed, Vyazovskiy et al. (2011) found that local use-dependent sleep (i.e., periods of neuronal silence) during wakefulness could be promoted by a repeated and intensive neuronal activity that, according to the ATP-cytokine-adenosine mechanism (Krueger et al., 2008, 2013), leads to an increased production of sleep regulatory substances. Many of these substances are involved in the homeostatic regulation of SWA, the most prominent local and use-dependent component of NREM sleep plasticity, as proposed by the synaptic homeostasis hypothesis (SHY) (Tononi and Cirelli, 2014). SHY claims that sleep would be necessary to reset the capacity to learn, saturated at neuronal level by waking-learning plasticity, so that the fundamental function of sleep may be the consolidation of memory. Moreover, SHY assumes that memory consolidation cannot be provided by wakefulness because it requires environmental disconnection (Tononi and Cirelli, 2014). Our findings indicate that specific networks, directly involved in learning-dependent plasticity processes and saturated by overlearning, may go off-line also during wakefulness.

At variance with previous human studies on local sleep in wake, in which the task merely involved visuomotor, attentional or categorization functions (Hung et al., 2013; Bernardi et al., 2015; Nir et al., 2017), in the present work we differentiated local use-dependent hd-EEG changes during an intensive spatial navigation task, that required a previous learning phase (formation and use of a cognitive map of a virtual city for wayfinding), from those of a control task without learning, that involved only the motor and procedural components of spatial navigation (directional arrows to be followed in different virtual environments without landmarks). This wake manipulation induced the increase of a specific index of learning-dependent plasticity, namely delta activity (Huber et al., 2004, 2006; Vyazovskiy et al., 2008) rather than theta (5–9 Hz), the typical EEG marker of sleepiness (Finelli et al., 2000). Theta and delta activity are claimed as the neural correlates of good spatial navigation ability (Watrous et al., 2011, 2013), however in the present study only delta activity was specifically associated to the spatial navigation task (wayfinding) that required learning, during which delta activity gradually became more intense across time, evidence of its use-dependent nature. During the control task, instead, delta and theta activity were prominent exclusively at the end (last quartile), possibly due to sleepiness (Finelli et al., 2000). While for theta no topographical differences were found between tasks, delta activity of the Wayfinding task was significantly higher than that of the Control task, as highlighted by source reconstruction analysis, that showed that delta activity was localized in a larger number of spatial navigation-related regions during Wayfinding than Control task, further supporting the relationship between delta activity and spatial navigation learning.

### Difference between local sleep in wake and sleepiness

The specificity of these effects of wayfinding on local sleep in wake is further highlighted by the subjective sleepiness and objective vigilance measures (psychomotor vigilance task, PVT). Although after both tasks (Wayfinding and Control) participants became more sleepy, subjective sleepiness did not differ between them at the end of the protocols. Therefore, the local increase of delta activity during Wayfinding cannot be merely accounted for by the increase of sleepiness over time. Moreover, PVT metrics not only resulted unaffected by the execution of both tasks, but these also did not differ between them at the end of the experimental protocols, as well as the relative delta and theta EEG activity. This is probably due to the fact that our experimental design did not employ sleep restriction/deprivation, at variance with recent studies on local sleep in wake (Hung et al., 2013; Bernardi et al., 2015; Nir et al., 2017), in which visuomotor and attentional performance was profoundly affected. Our approach was rather based on the manipulation of the task characteristics (with/without learning), maintaining the same (long) time-on-task, in order to induce local sleep processes during wake even in a circadian phase favorable to alertness. In fact, according to recent evidence, not only homeostatic sleep pressure (Tononi and Cirelli, 2014) but also circadian rhythms affect brain locally (Muto et al., 2016). To avoid both the circadian influence on sleep pressure and the restriction of sleep duration imposed by extended wakefulness protocols on neurobehavioral performance (Hung et al., 2013; Bernardi et al., 2015; Nir et al., 2017), both experimental tasks were scheduled in the morning and performed in a proper vigilance window, namely from ~9:00 to ~13:00 (Van Dongen and Dinges, 2005; Muto et al., 2016).

### Relation between delta activity, time on task-effect and performance failures

Time-on-task effect, namely the decline in performance that increases as a function of the duration of a cognitive task, should be the result of an intensive repeated use of specific neuronal groups engaged in the task at hand (Van Dongen et al., 2011). For this purpose, we explicitly manipulated the Wayfinding task that, beyond the learning phase (formation of the cognitive map of a virtual city), was scheduled to run continuously, without any kind of rest breaks, and required the repetition of the same pool of retrievals (*overlearning*, Shibata et al., 2017). Despite the achievement of a stable performance after ~20 min (in line with Shibata et al., 2017) and many repetitions of the same retrievals, after about 40 min subjects began to make several errors that increased in the course of time. At the same time delta activity associated to errors and hits, at variance with theta activity, increased locally and progressively during this task. Errors occurred during well-learned retrievals and they were preceded and followed by hits. Therefore, we can exclude that errors could depend on a wrong or inadequate learning.

According to literature, low-frequency oscillations during spatial navigation are related to memory processing (Jacobs, 2013; Ekstrom and Watrous, 2014; Vass et al., 2016). As previously pointed out, the progressive increase of delta activity differentiates between Wayfinding and Control task: the increase of delta activity across time was specifically associated to the former, that is the spatial navigation task that required *learning*. These findings are further supported by source reconstruction, revealing that delta activity was mainly localized in the left parietal and bilateral frontal cortices, all regions engaged in memory-based spatial navigation tasks (Kravitz et al., 2011; Boccia et al., 2014; Ekstrom et al., 2014; Slone et al., 2016). Interestingly, the regions detected by source reconstruction have been recently linked to navigation in simple virtual environments (Slone et al., 2016), as if the intensive repetitions of the same retrievals had made the complex virtual city progressively more familiar. We cannot rule out the involvement of subcortical regions, as hippocampus, during both tasks, however they cannot be detected by hd-EEG. Although also theta was localized in the left parietal and bilateral frontal cortices, this occurred only at the end of the Wayfinding task. Thus, as mentioned above, theta source activity could be interpreted as an index of sleepiness.

As for delta activity, although hits and errors showed similar source activation, the correlations between delta source activity and behavioral outcomes allowed us to differentiate hits from errors. Indeed, only errors positively correlated with delta activity of the left parietal and right frontal cortices, whereas hits negatively correlated with delta activity in the bilateral frontal cortex. In other words, the higher the errors, the higher the delta in brain regions critically involved in navigation within recently learned environments like the precuneus and the inferior parietal lobule within the posterior parietal cortex (Boccia et al., 2014) as well as the right prefrontal cortex (e.g., Slone et al., 2016); moreover, the smaller the hits, the higher the delta in the bilateral frontal cortex, which might be congruent with top-down executive control of visuospatial processing by the prefrontal cortices (Kravitz et al., 2011).

Given that, we decided to investigate in the aforementioned brain regions (left parietal, right and left frontal) the individual *delta* waves of errors and hits, as the sign of local sleep in wake, according to our experimental hypothesis. In fact, performance errors during wake are associated with the negative peak of individual slow waves, that correspond to periods of neuronal silence (off-periods) of the detected waves (Vyazovskiy et al., 2011; Nir et al., 2017).

### Individual delta waves as indexes of local sleep in wake

From the analysis of the individual delta waves in wakefulness a core finding of the current study emerged, namely that performance impairments are specifically associated to periods of neuronal silence, possibly as the results of the synaptic saturation of spatial navigation circuits. Indeed, during the Wayfinding task, we found that off-periods of individual delta waves were higher, in terms of number, and deeper, in terms of negative amplitude, during errors than hits. Thereby, these errors might be considered the behavioral counterpart of periods of neuronal silence due to use-dependent processes, a sign of learning-dependent plasticity, only apparently maladaptive. In other words, these off-periods may allow a brief rest to the overloaded networks, and they could be related to the first steps of the memory consolidation processes. As a (negative) consequence, it is possible that in these moments subjects could not retrieve the best path they learned and reached previously, choosing instead old (used before performance stabilization) and unusual alternative paths because of the synaptic overload in the specific spatial navigation circuits. Thereby, it would be interesting to investigate changes of dynamic functional connectivity in the time window between errors and the subsequent hits. Successful spatial navigation in recently learned environments indeed engages a complex network (Boccia et al., 2014), and errors could represent the behavioral output of the shutdown of the functional connectivity within this network.

## Conclusions

The proposed “learning-related use-dependent local sleep model” suggests a pathway in which even performance failures (errors) could be functionally linked to learning-related plasticity, when they result from local use-dependent sleep periods (off periods) in wake. Local sleep in wake can lead to errors, which may be the dangerous but necessary consequence of learning-dependent synaptic overload, possibly initiating local recovery and consolidation processes. In a similar way, even if sleep represents a significant danger to survival from an evolutionary perspective, memory consolidation attributes to it a universal and essential function. However, future studies are needed to further elucidate the functional implications of the described off periods in wake for performance recovery or memory consolidation.

## Author contributions

AQ, FZ, GC, and MF: designed the study, interpreted the data and wrote the manuscript; AQ, and FZ: carried out the experiments and analyzed the data. All of the authors participated in drafting the work and agreed on the final version of the manuscript.

### Conflict of interest statement

The authors declare that the research was conducted in the absence of any commercial or financial relationships that could be construed as a potential conflict of interest.
