# Supplementary material for: Local Use-Dependent Sleep in Wakefulness Links Performance Errors to Learning
Source: Front Hum Neurosci. 2018 Apr 3;12:122. doi: 10.3389/fnhum.2018.00122 (PMC5891895; doi:10.3389/fnhum.2018.00122)
Supplement: Supplementary file 1 [file DataSheet1.pdf]

Supplementary Material

Local use-dependent sleep in wakefulness links performance errors to learning

Angelica Quercia [1*], Filippo Zappasodi [1,2], Giorgia Committeri [1,2], Michele Ferrara [3].

[1] Department of Neuroscience, Imaging and Clinical Sciences, "G. d'Annunzio" University of Chieti-Pescara, Chieti, Italy

[2] Institute for Advanced Biomedical Technologies (ITAB), " G. d'Annunzio " University of Chieti-Pescara, Chieti, Italy

[3] Department of Biotechnological and Applied Clinical Sciences, University of L'Aquila, Coppito (AQ), Italy

*Corresponding author: Angelica Quercia, Ph.D.

Department of Neuroscience, Imaging and Clinical Sciences, via Luigi Polacchi 11

"G. d'Annunzio" University of Chieti-Pescara,

66013 Chieti, Italy

Phone: +39 0871 3556901/02

angelica.quercia@gmail.com

Supplementary Table 1. Inclusion criteria.

To rule out Depression and Anxiety, BDI-II (Sica et al., 2006) and Stay-Y2 (Spielberg et al., 1983); to include subjects with a good sleep quality PSQI (Curcio et al., 2013), without excessive daytime sleepiness ESS (Vignatelli et al., 2003), with intermediate chronotype MEQ (Horne and Ostberg, 1976) and without sleep disorders SDQ (Violani et al., 2004); the type of orientation skills employed by subject's was assessed by QOS (Pazzaglia et al., 2000), a self-rating questionnaire, built on the basis of the landmark-route-survey (LRS) navigation model (Wolbers and Hegarty, 2010).

**Questionnaires**	**mean ± S.D.**	**Cut off**
**Psychiatric disorders**		
BDI-II (Beck Depression Inventory)	2,4 ± 2,66	<13
Stai-Y2 (Trait Anxiety Inventory)	32,9 ± 4,25	<40
**Sleep**		
PSQI (Pittsburgh sleep quality index)	2,45 ± 1,14	<5
ESS (Epworth sleepiness scale)	4,95 ± 2,18	<10
MEQ (Morningness eveningness questionnaire)	54,45 ± 4	42-58
SDQ (Sleep Disorders Questionnaire)	-	n1
**Spatial skills**		
QOS (Spatial Orientation Questionnaire)	56 ± 7,15	>43

Supplementary Table 2. Pilot study of the Wayfinding task

To test the new 3D virtual environment, an independent sample of 22 male subjects performed the Wayfinding task. So, they had to explore freely the environment until they had formed a mental representation of it, including the sixteen identifiable landmarks; then, as soon as they had spontaneously stopped, they were asked to report the locations of the 16 different landmarks on the schematic map. Participants explored the environment for further sessions until they reached an accuracy of 100% for two consecutive maps.

	**mean ± S.D.**
Subjects Age (22 males)	22,86 ±2,86 (years)
Mean time of the first exploration of the environment (spontaneously stopped)	13, 35 ± 3,13 (min)
Mean duration of the Learning phase	37,40 ±3,08 (min)
Mean number of maps to form the mental representation of the environment (100% accuracy)	2,59 ±0,65

Supplementary Table 3. Sleep Efficiency.

To avoid any kind of sleep debt, Sleep Efficiency above 85% was required 1 week and the night before each experiment.

**Sleep Efficiency 20 subjects (%)**	**mean ± S.D.**	**Cut off**
Mean (1 Week) Pre-Wayfinding Task	89,35 ± 3,70	>85
Mean of the Night before Wayfinding Task	90,02 ± 3,52	>85

**Sleep Efficiency 9 subjects (%)**	**Median (min, max)**	**Cut off**
Mean (1 Week) Pre- Wayfinding Task	89,06 (85,43; 98,00)	>85
Mean (1 Week) Pre-Control Task	91,57 (85,09; 97,42)	>85
Mean of the Night before Wayfinding Task	90,36 (85,63; 96,38)	>85
Mean of the Night before Control Task	91,20 (85,52; 95,06)	>85

Figure S1. Trend analysis

A

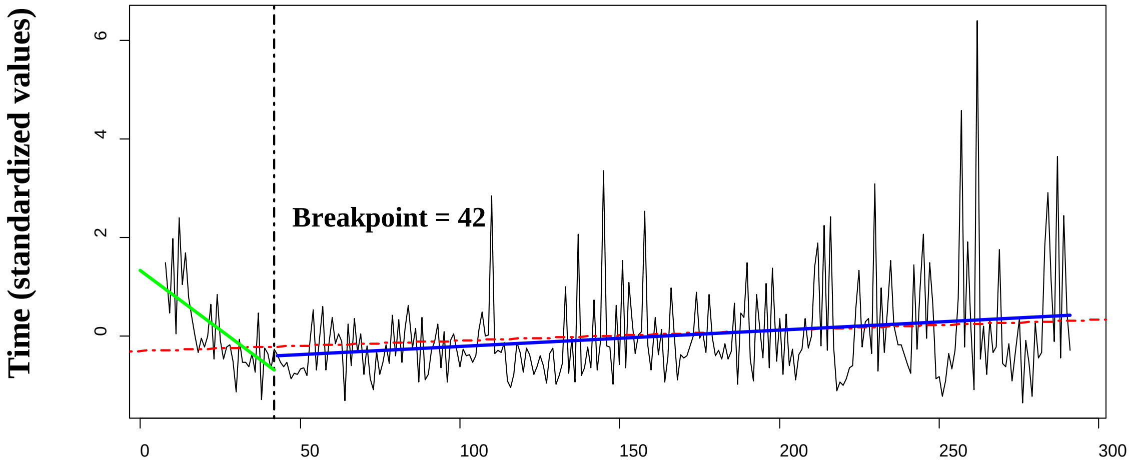

B

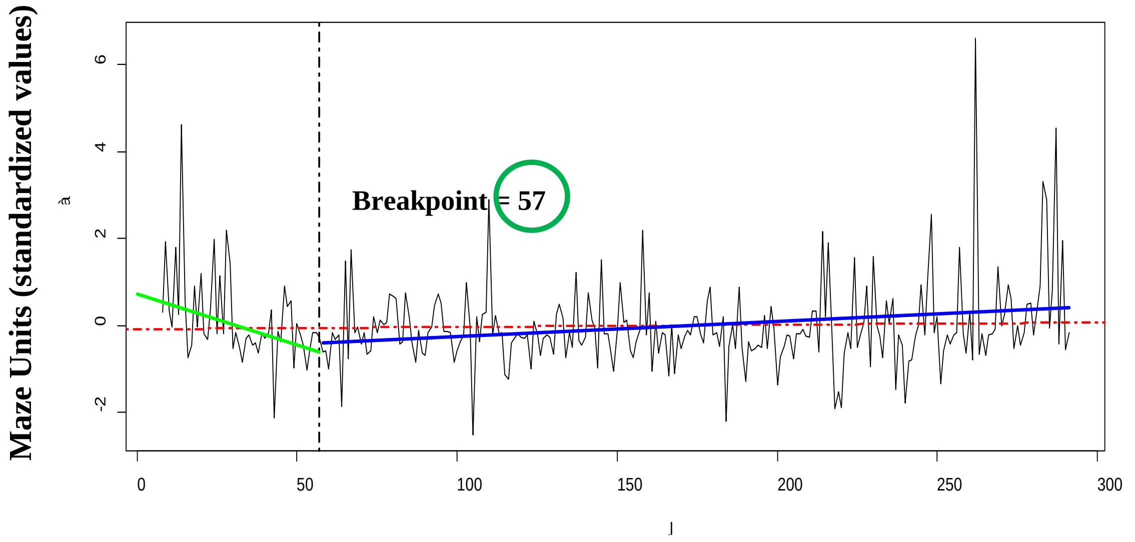

Supplementary Figure 1. Representative trend analysis (one subject) for the time (A) and units (B) of the test phase (sequence of retrievals); the vertical line indicates the first breakpoint, retrieval number 42 and retrieval number 57 for time and units sequence, respectively; vertical line indicates the breakpoint, the red lines are the regression line, the green and the blue ones refer to the piecewise regressions lines calculated on the basis of the first breakpoint identified; the green circle (B) indicates the breakpoint chosen (the greater one) for further analyses.

Supplementary Video 1. Video recording of a representative Error.

This movie shows an Error during the retrieval “Town Hall – Flower Shop”, the same displayed as graphic layout in Figure 1B. Although it was the forty-ninth time that the subject had repeated this retrieval, with several previous successes, during the route he got lost, choosing wrong destinations. Interestingly, along the route, he did not go by chance, but he rather chose the destinations of previous retrievals, overlooking other destinations. Note that the subject was clearly behaviorally awake throughout the retrieval.
